# Micronutrient Deficiencies in Obese Patients and Risk of Postoperative Fistula: A Forgotten Link in Bariatric and Metabolic Surgery

**DOI:** 10.3390/nu18071131

**Published:** 2026-03-31

**Authors:** Ludwig Alvarez-Cordova, Victoria Gonzalez, Facundo Saettone, María Sol Barry, Laura Verónica Godoy, Julieta Siman, Natalia Llobera, Melannie Toral-Noristz, Sebastián Chapela

**Affiliations:** 1 Maestría de Nutrición y Dietética, Facultad de Ciencias de la Salud, Universidad de las Américas, Quito 170124, Ecuador; 2 Unidad de Soporte Metabólico y Nutricional, Sanatorio Allende, Córdoba X5000BFB, Argentina; vgonzalez@sanatorioallende.com; 3 Facultad de Ciencias de la Salud, Universidad Católica de Córdoba, Córdoba X5000IYG, Argentina; 4 Servicio de Alimentación, Hospital Nacional “Profesor Alejandro Posadas”, Buenos Aires 1684, Argentina; facundo.saettone1@gmail.com; 5 Servicio de Endocrinología, Metabolismo, Nutrición y Diabetes, Hospital Británico de Buenos Aires, Buenos Aires 1280, Argentina; mbarry@hbritanico.com.ar; 6 Área de Nutrición Clínica, Hospital Británico de Buenos Aires, Buenos Aires 1280, Argentina; lauvgodoy@gmail.com; 7 Servicio de Nutrición y Alimentación, Hospital Central de Mendoza, Mendoza 5500, Argentina; nutricion.julietasiman@gmail.com; 8 Equipo de Soporte Nutricional, Hospital Británico de Buenos Aires, Buenos Aires 1280, Argentina; naty.llobera@gmail.com (N.L.); sebachapela@gmail.com (S.C.); 9 Escuela de Medicina, Universidad de Especialidades Espíritu Santo, Samborondón 092301, Ecuador; melannietoral@gmail.com; 10 Departamento de Bioquímica, Facultad de Ciencias Médicas, Universidad de Buenos Aires, Buenos Aires C1428EGA, Argentina

**Keywords:** obesity, vitamin, trace elements, anastomotic leakage

## Abstract

Micronutrient deficiencies are commonly observed in patients with obesity and may persist or worsen following bariatric and metabolic surgery. Emerging evidence suggests that micronutrients play a fundamental role in tissue repair, collagen synthesis, immune function, and inflammatory regulation processes that are critical in postoperative healing. Therefore, deficiencies in these nutrients could be pivotal in understanding and preventing postoperative complications. However, the potential link between preoperative micronutrient status and the development of postoperative complications, such as anastomotic or gastric fistula, remains underexplored. This narrative review aims to investigate the correlation between specific micronutrient deficiencies (e.g., vitamin C, zinc, selenium, vitamin A, and iron) and the incidence of fistula after bariatric surgery. We will discuss the underlying biological mechanisms, clinical evidence, and possible preventive strategies, including preoperative screening and targeted supplementation. Our aim is to highlight the often-overlooked micronutrient deficiency as a risk factor in patients undergoing bariatric surgery, both in the pre- and postoperative periods, and to propose a more comprehensive approach to patient assessment and management.

## 1. Introduction

Obesity is a global problem, affecting all social classes. Its incidence is increasing over time [1,2,3,4]. This pathology is associated, among others, with an increased incidence of diabetes, cardiovascular diseases, liver diseases, sleep apnea, and various types of cancer [5,6,7,8]. Obesity treatment involves a combination of lifestyle modifications, pharmacotherapy, and surgical interventions tailored to individual needs and disease severity [8,9,10]. Pharmacological advances, including glucagon-like peptide-1 (GLP-1) receptor agonists like semaglutide and dual gastric inhibitory polypeptide (GIP) GIP/GLP-1 receptor agonists, such as tirzepatide, have demonstrated significant weight loss effects exceeding 10–20%, with outcomes similar to bariatric surgery [11,12]. Bariatric surgery remains the most effective treatment for severe obesity, typically resulting in durable weight loss of about 25% and rapid improvement in obesity-related complications, but requires specialized multidisciplinary care [13].

Surgical techniques for this type of metabolic surgery include sleeve gastrectomy (SG) and Roux-en-Y gastric bypass (RYGB) [14,15,16,17]. While bariatric surgery is a successful strategy, this procedure is not without complications [14]. Nutritional deficits, acid reflux, anastomotic stenosis, gallstone disease, leaks, fistulas, and weight gain are some of the procedure’s consequences [18]. Many of these complications have a long convalescence period, even requiring long periods of home parenteral nutrition [19]. The incidence of complications related to the anastomosis can reach 1% [14,20]. According to various studies, age and male sex are risk factors for anastomotic leaks [20,21].

However, vitamin and trace element deficiencies are highly prevalent in these patients, both before and after surgery [22]. These deficiencies, in various models and other pathologies, are associated with impaired wound closure, altered collagen formation, and increased oxidative stress [22,23]. The significance of this review lies in its integrative approach, moving beyond traditional surgical risk factors to focus on the metabolic environment of the patient. While the existing literature often treats micronutrient deficiencies and surgical complications as separate entities, this work contributes to current knowledge by synthesizing the biochemical pathways through which specific deficits directly impair anastomotic healing. This perspective is crucial for clinical practice, as it provides a theoretical and practical framework for surgeons and nutritionists to transition from generic supplementation to a precision-based “metabolic optimization” before surgery. Despite the high prevalence of micronutrient deficiencies in candidates for bariatric surgery—reaching up to 85.5% in some cohorts—research has traditionally focused on technical or demographic risk factors for postoperative fistulas [24,25,26]. However, the biological role of micronutrients as essential “builders” in collagen synthesis, immune response, and oxidative stress regulation suggests they are critical yet overlooked determinants of anastomotic integrity. This review seeks to answer the following question: To what extent do pre-existing and postoperative micronutrient deficiencies compromise the biological stages of healing and increase the risk of fistula? Consequently, the objective of this narrative review is to evaluate the correlation between specific micronutrient deficits and fistula incidence, analyze the underlying biological mechanisms, and propose evidence-based integrative strategies for preoperative screening and targeted supplementation to mitigate these risks.

## 2. Materials and Methods

For this narrative review, significant articles were taken into consideration. The search was done via PubMed using a combination of related search terms, including “obesity”, “micronutrients”, “vitamins”, “anastomotic leakage”, “oligoelements”, and “trace elements”. According to their pertinence, titles and abstracts of the identified articles were examined by research team members and were selected for full review if the authors agreed. Additionally, the references of identified articles were also analyzed so that these publications could be included. The time frame used for the search was between 2015 and 2025. However, if any author considered an article pivotal for the understanding of an idea, it was included. Once all the articles were identified, the research team categorized each article based on the relationship between micronutrient deficit and postoperative fistula. Finally, relevant articles on the topic were selected, excluding case reports, case series, and expert opinions.

## 3. Micronutrient Deficiencies in Obesity and Bariatric Surgery

Obesity is linked to a higher incidence of nutritional deficiencies [26]. Obesity-specific metabolic, inflammatory, and nutritional variables all contribute to this condition [26,27]. Although bariatric surgery is the best therapy for severe obesity, it is associated with an increased risk of nutritional deficiencies due to reduced intake, malabsorption, and anatomical changes, as well as alterations in gastrointestinal hormones such as ghrelin, glucagon-like peptide-1 (GLP-1), and peptide YY (PYY).

### 3.1. Baseline Micronutrient Deficiencies in Obese Individuals

The most prevalent and most reported deficit in obesity is vitamin D insufficiency [28,29,30]. Observational studies show that vitamin D insufficiency is 35% more common in people with obesity than in people of normal weight [31]. In a cross-sectional study of candidates for bariatric surgery, 57% of individuals were found to have vitamin D deficiency, defined as 25(OH)D < 20 ng/mL (50 nmol/L) [32]. There is a negative correlation between low serum 25(OH)D levels and obesity, according to observational investigations [33]. This indicates that the majority of obesity indices, including body mass index (BMI), total fat mass, subcutaneous and visceral adiposity, and waist circumference, have an inverse relationship with plasma 25(OH)D [34]. In a meta-analysis with 257,699 participants, dose–response analysis showed that every 25 nmol/L rise in blood vitamin D was linked to an 8% decreased risk of abdominal obesity [35].

Low blood retinol concentrations and vitamin A deficiency have been reported in people with obesity; however, they are less prevalent than vitamin D deficiency [28,36]. Vitamin A deficiency in people with obesity can vary from 15% to 50%, particularly when hyperglycemia and a worse metabolic profile are present [28]. In a cross-sectional study with 267 obese patients (BMI 43.2 kg/m^2^ (standard deviation: 6.2)) who were candidates for bariatric surgery, micronutrients were assessed before surgery [36]. It was observed that 16.9% had low retinol, or vitamin A deficiency, and their levels dropped as BMI increased [36]. The patients also had low levels of 25(OH)D, magnesium, phosphate, and iron [36]. Also, in an observational trial, preoperative body mass index (BMI) and baseline retinol had a negative relationship (R = −0.15, *p* = 0.007) [37]. Six months after surgery, both overall vitamin A deficiency (oVAD) and moderate vitamin A deficiency (mVAD) peaked (33% vs. 15%, *p* < 0.005; 12% vs. 4%, *p* = 0.0004, respectively) [38]. At 24 months, oVAD was still higher (22% vs. 15%, *p* = 0.03). oVAD was higher with gastric bypass (GB) at 6 months (39% vs. 28%, *p* = 0.001) and also 12 months after surgery (26% vs. 17%, *p* = 0.04) compared to SG, and mVAD was higher with GB at 6 months (18% vs. 6%, *p* < 0.0005) [37].

Another common vitamin deficit is associated with vitamin C. Reduced fruit and vegetable consumption, which is frequent with obesity, and elevated oxidative stress have been linked to low plasma levels of vitamin C [39]. According to a narrative review, people with obesity frequently have lower levels of vitamin C as well as other fat-soluble and water-soluble vitamins, including folate and B-complex vitamins [39].

Not only is obesity associated with vitamin deficiencies, but also with trace element deficiencies. In many cases, people with obesity-related metabolic issues often experience a lack of iron [34,40,41]. This is largely because obesity is associated with higher levels of hepcidin, a protein that plays a role in regulating iron, and with ongoing low-level inflammation [34,40,41]. Studies show that between 6% and 45% of obese individuals (median BMI 46.3 kg/m^2^ ± 6.9) who undergo bariatric surgery suffer from iron deficiency or iron-deficiency anemia, which is much more common than in the general population, where these conditions occur in about 6% to 7% of people [34]. Among women, iron deficiency is found to be 9%, and the rate of iron-deficiency anemia is around 7%, increasing as body mass index rises [34]. Women are more prone to developing iron deficiency compared to men [34]. In pre-bariatric morbid obesity (mean BMI: 44 ± 9 kg/m^2^), only 9.6% had ferritin <15 µg/L, compared to other studies where the percentage of iron deficiency is higher [38].

Zinc deficiency is also commonly reported in individuals with obesity. Because of its functions in the immune system, wound healing, and energy metabolism, zinc is particularly important [42]. In women with obesity, zinc deficiency has been reported in approximately 20% of cases. Low serum zinc concentrations are associated with poor antioxidant defense [42]. Additionally, it plays a major role in the development of several cancers linked to obesity as well as acute and chronic liver disorders [42]. Finally, selenium is a vital trace element that helps lower inflammation and functions as an antioxidant defense [43,44,45]. The primary change in selenium nutritional status under situations of excess adiposity is a reduction in glutathione peroxidase activity, particularly in obese individuals [44]. The existing investigations conclude that individuals with obesity exhibit changes in selenium biomarkers or suboptimal values, despite the lack of a defined prevalence figure [44].

The need for substances with anti-inflammatory and antioxidant qualities to assist in managing metabolic problems linked to obesity is increased by oxidative stress and chronic low-grade inflammation [24]. Inflammation raises the risk of micronutrient deficiency, and obesity is a chronic pro-inflammatory condition [24]. Despite consuming a lot of calories, individuals with obesity often have baseline shortages in several micronutrients. Inappropriate weight-loss plans, altered micronutrient metabolism, bioavailability, and distribution, persistent inflammation, and decreased sun exposure (for vitamin D) are all linked to poor diet quality [24].

### 3.2. Postoperative Micronutrient Challenges

Nutritional deficiencies during the postoperative period are dependent on the type and technique of surgery (malabsorptive or restrictive), which can result in changes in absorption and digestion as well as postoperative complications like food intolerance, nausea and vomiting, altered eating habits, small intestinal bacterial overgrowth (SIBO), and noncompliance with dietary and supplement recommendations [46,47,48]. The risk of dietary inadequacies is greatly increased by this [49]. Due to limitations, malabsorption, and, most importantly, the modification of the gut–brain axis, the Roux-en-Y gastric bypass reduces total calorie intake by rerouting absorbed nutrients to the jejunal limb and diverting the duodenum and proximal jejunum [50]. Sleeve gastrectomy is a restrictive procedure whose effectiveness is mediated by the decrease in gastric capacity and neurohormonal alterations [32,50]. Because the diverted small intestine is longer, biliopancreatic diversion, both with and without duodenal switch, increases the risk of nutritional deficiencies [32,50].

**Figure 1:**
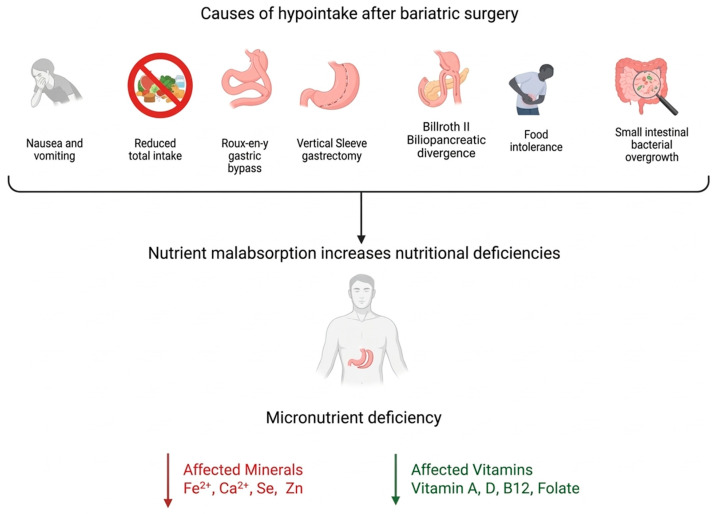
Nutritional deficiencies post-bariatric surgery. Bariatric surgical procedures produce sustained beneficial effects on weight loss. The causes of nutrient deficiencies in the postoperative period become clear when considering the areas of the intestine responsible for the absorption of both micronutrients and macronutrients and the portion of the intestine bypassed or removed in each bariatric procedure. Furthermore, complications of the intervention, such as food intolerance, the onset of gastrointestinal symptoms, bacterial overgrowth, and inadequate diets, must also be considered. This leads to an increased risk of malnutrition and the resulting nutritional deficiencies experienced by patients undergoing bariatric surgery [32,50].

The mechanisms that perpetuate these deficiencies are related to reduced total intake and the resection of key intestinal segments for nutrient absorption, such as the duodenum and jejunum, the main sites for the absorption of iron, zinc, and calcium [22,50]. Fluctuations in gastric pH and diminished intrinsic factor secretion compromise the efficiency of vitamin B12 absorption [50]. Fat malabsorption secondary to surgical procedures compromises the absorption of fat-soluble vitamins [50]. Figure 1 summarizes changes in micronutrients after surgery.

Compared to the preoperative situation (57%), the postoperative prevalence of vitamin D insufficiency revealed rates as high as 64% [33,51]. These findings demonstrated that all postoperative bariatric surgery patients should have their 25(OH)D levels regularly measured and that they need to take particular regular vitamin D supplements [33,51]. Although it is still given in various forms and dosages, it is now generally acknowledged that patients need vitamin D supplements following bariatric surgery [33,51]. Regardless of the type of surgical intervention and treatment timing, postoperative vitamin D supplementation doses ≥2000 IU/day lead to lower rates of vitamin D insufficiency (as defined by the 30 ng/mL criterion only) than doses <2000 IU/day [33,51].

In an observational study of patients undergoing bariatric surgery, the prevalence of vitamin B12 deficiency decreased from 13.5% at the time of surgery to 2.0% at the early postoperative follow-up, before increasing again to approximately 12% at 18–24 months, and was influenced by preoperative vitamin B12 status [52]. Limited use of micronutrient supplements and poor absorption of vitamin B12 may be the cause of this deficit [22,52]. Through a number of processes, including gastric achlorhydria, reduced intrinsic factor production, and bacterial overgrowth in the ileum, bariatric surgery reduces the absorption of vitamin B12 from food [22]. The postoperative vitamin B12 insufficiency is only partially explained by these processes, which also lead to iron and folate malabsorption [22].

Iron deficiency is seen in 18.0–53.3% of patients throughout the long-term postoperative phase, and iron deficiency anemia affects 52–54% of patients [22,53]. Higher risks of postoperative complications are linked to iron deficiency anemia [22]. Compared to males receiving gastric sleeve and gastric banding surgeries, women undergoing Roux-en-Y gastric bypass (RYGB) procedures are twice as likely to get anemia [40]. Reduced iron intake from poor absorption, poor tolerance to meals high in iron, poor patient adherence to iron-containing medications, and decreased hydrochloric acid secretion as a result of the surgical procedure all contribute to iron insufficiency following bariatric surgery [40].

Zinc solubilization and absorption are further reduced by the decrease in stomach acid production [22,54]. Zinc deficiency is quite prevalent in bariatric patients due to these changes, which are made worse by dietary restrictions following surgery [22,54]. Alopecia, delayed wound healing, and immunological issues are among the nonspecific signs of this insufficiency [22,54]. Also, copper deficiency after surgery was associated with neurological illnesses, as reported in a growing number of reports of neurological problems after the procedure [55].

### 3.3. Importance of Preoperative and Postoperative Nutritional Monitoring

Preoperative screening and correction, periodic biochemical monitoring to determine personalized supplements, nutritional education, and monitoring of adherence to both supplementation and nutritional treatment are key challenges in preventing and early diagnosing of deficiencies [49,56,57,58,59]. In order to treat the frequent nutritional deficiencies in individuals undergoing bariatric surgery, preoperative micronutrient supplementation is crucial [49,56,57,58,59]. Malnutrition affects up to two-thirds of patients who are scheduled for bariatric surgery [49,56,57,58,59]. It is necessary to address deficiencies in important nutrients such as vitamin D, calcium, thiamine, vitamin B12, folate, iron, zinc, copper, and fat-soluble vitamins [49,56,57,58,59]. To maximize recovery, preoperative supplements should start eight to ten weeks before surgery [49,56,57,58,59].

To lower the risk of osteoporosis and fractures, which are known to rise in individuals after bariatric surgery, calcium and vitamin D levels should be routinely evaluated [32,58,60]. One of the micronutrients that should be measured is vitamin A, particularly in the first year following a pancreaticoduodenectomy (PD) with or without duodenal switch or Roux-en-Y gastric bypass (RYGB) [32,58,60]. Patients with coagulopathy, liver disease, and neuromuscular impairment should have their vitamins E and K examined [32,58,60]. To improve iron absorption, vitamin C supplements may be taken into consideration [32,58,60]. Since vitamin B12 deficiency can develop years after bariatric surgery and manifest as megaloblastic anemia and neuropathy, among other symptoms, vitamin B12 monitoring should be done for all patients before and at least once a year, preferably sooner following surgery [32,58,60]. 

For comprehensive knowledge, screening instruments and laboratory testing should be incorporated into nutritional status assessments [22]. In order to avoid nutritional shortages following bariatric surgery, immediate supplementation is essential [22]. Depending on the type of surgery, it is advised to begin multivitamin and mineral supplementation right away [22]. Over time, adherence to supplementation regimens declined dramatically, raising the possibility of inadequacies [22]. It is advised to take multivitamins throughout the rest of one’s life and to modify supplementation according to personal needs [22]. It is recommended to conduct tests every three months for the first year and then once a year after that [22]. Emphasis should be placed on the significance of precise preoperative evaluation of dietary deficits, postoperative monitoring, and suitable replacement [38]. Healthcare personnel need to be made aware that people with morbid obesity may exhibit clinically significant nutritional deficits despite their high calorie intake [38]. Preoperative management of these impairments may enhance the prognosis after surgery and lessen postoperative metabolic problems [38]. 

## 4. The Biology of Healing: Why Micronutrients Matter

### 4.1. Collagen Synthesis and Tissue Repair

Wound healing is a process involving consecutive and overlapping stages, such as homeostasis, inflammation, proliferation or formation of new tissue, and remodeling [61,62]. Three days post-injury, macrophages replace neutrophils, initiating granulation tissue penetration. Macrophages promote angiogenesis and extracellular matrix (ECM) formation, facilitate repair, and remove debris. Epithelialization simultaneously restores epidermal thickness via collagen fibers [63]. Collagen formation is mainly carried out by fibroblasts in the dermis [63], replacing the fibrin clot with a stable collagen matrix [61].

Vitamin C is vital for wound healing: it aids fibroblast migration, acts as a cofactor for procollagen hydroxylation to collagen, and stimulates collagen mRNA production [63,64,65]. Deficiency leads to poor healing due to inadequate collagen formation [61]. Studies support its efficacy: one randomized controlled trial (RCT) with patients with foot ulcers (mean BMI 26.2 kg/m^2^; 95% IC: 2.1) showed significantly better healing with vitamin C, correlating baseline serum levels negatively with ulcer size (r = −0.622, *p* < 0.05) [66]. The enriched formula led to a significantly greater mean reduction in pressure ulcer area (57% vs. 33% at week 8, *p* < 0.02; and 72% vs. 45% at week 12, *p* < 0.005), suggesting the key was the combined effect of the enriched formula [67]. Another multicenter study with patients with pressure ulcers (mean BMI 22.9 ± 5.9 kg/m^2^) confirmed that an oral supplement with protein, arginine, zinc, and vitamin C significantly reduced pressure ulcer size and improved wound characteristics [68].

Zinc is vital for wound healing as a cofactor for DNA/RNA polymerase, supporting DNA/protein synthesis and cell proliferation. Deficiency delays healing by decreasing fibroblast proliferation and collagen synthesis [69]. Zinc plays a crucial role in all wound-healing stages by affecting cell behavior and regulating enzyme activity [70]. Zinc metalloenzymes, such as matrix metalloproteinases (MMPs), are key for ECM remodeling and mark early angiogenesis, inflammation, and connective tissue proliferation [71]. Alkaline phosphatase, another zinc enzyme, is also an early marker of post-inflammatory angiogenesis and connective tissue proliferation [70]. Zinc promotes hemostasis by enhancing platelet activity and aggregation, amplifying responses to agonists, and stimulating aggregation at high concentrations [72]. Deficiency prolongs bleeding time by altering coagulation and fibrin cascades [73]. Therefore, zinc regulates platelet aggregation, coagulation, and fibrin network formation to support hemostasis [74]. Zinc levels increase significantly (15–20%, up to 30%) during the initial inflammation and intensive granulation tissue formation phases of wound healing [75]. The subsequent decrease in zinc during the final stages (10–21 days) correlates with reduced mitotic activity and wound maturity [71]. During the proliferation phase, zinc-finger, X-linked protein (ZFX) promotes keratinocyte proliferation and migration [76]. This phase also involves re-epithelialization, where endothelial cells migrate and proliferate to form new blood vessels, supplying essential oxygen and nutrients [70]. In the remodeling phase, wound epithelialization restores dermal strength, a process involving zinc-dependent MMPs for structural organization and regeneration of the dermis and epidermis [77].

Likewise, copper is also essential for wound healing, playing a key role in the induction of vascular endothelial growth factor (VEGF), angiogenesis, and the expression and stabilization of extracellular skin proteins [78]. Platelet-derived growth factor (PDGF) requires copper for signaling and acts as an important angiogenic factor during the proliferative phase [78,79]. Copper ions also regulate the activity and expression of MMPs, crucial proteases that remove damaged proteins, facilitate cell migration, and remodel granulation tissue, vital for ECM remodeling and the proliferative phase of wound healing [79]. Copper nanoparticles also induce the expression of molecules, such as integrins, fibrinogen, and collagen, which are involved in the formation of the ECM and are crucial for healing [80]. Copper promotes regeneration and improves skin quality. It also reduces the duration of the inflammatory phase, allowing for a faster transition to the final phase of angiogenesis and the production of new collagen [80].

Collagen is rich in glycine (one-third), proline, and its derivative hydroxyproline (23%) [81]. Prolyl hydroxylase, requiring vitamin C, converts proline to hydroxyproline [81]. Arginine is conditionally essential for wound healing during stress, as endogenous synthesis is insufficient [81]. Arginine is a substrate for arginase and nitric oxide synthase (NOS) [82]. NOS produces nitric oxide (NO), a potent vasodilator that increases vascular permeability, mediates immune responses, inhibits platelet aggregation, acts as a cell signaling molecule, and is cytotoxic/toxic to bacteria in wounds [82]. Fibroblast-derived NO induces collagen, while its second pathway suppresses production by increasing arginase activity [82]. Arginase converts arginine into proline (a key collagen component) and ornithine, a precursor to putrescine and polyamines needed for DNA replication [82]. A systematic review and meta-analysis found that arginine and glutamine supplements impact wound healing; arginine specifically increased hydroxyproline, suggesting higher collagen deposition [83]. However, another systematic review on chronic wound healing showed arginine alone was not a determining factor [84]. Its efficacy may rely on combinations with proteins and antioxidant micronutrients, while formulas with arginine, glutamine, and β-hydroxy-β-methylbutyrate were ineffective [84]. Conversely, a meta-analysis on pressure ulcers revealed that arginine supplementation significantly improved healing, with a higher dose of 15 g/day being most beneficial [85].

Micronutrients are not only essential for the healing of skin wounds but are also necessary to maintain the integrity of anastomoses. A case–control study investigated the effect of oral vitamin C administration on anastomotic resistance in rats [86]. They observed that the rats that received vitamin C had higher anastomotic bursting pressure, with the most significant difference occurring 28 days postoperatively when the rats treated with vitamin C had considerably higher tension (*p* = 0.037) [86]. In addition, the vitamin C group showed a higher collagen concentration (2.46 ± 0.23) compared to the control group (1.83 ± 0.14) (*p* = 0.021), demonstrating that oral administration of vitamin C significantly increases anastomotic resistance [86]. On the other hand, zinc is vital for DNA and protein synthesis as it influences cell proliferation and inflammatory response, as well as improving antioxidant defenses, and its deficit can increase oxidative stress, further compromising wound integrity [87]. In a study in which daily administration of zinc and vitamin C supplements was given to rabbits with peritonitis undergoing colonic anastomosis, the findings suggest a positive relationship between supplementation and improved anastomotic integrity through a significant difference in collagen density, neovascularization, and the presence of fibroblasts, contributing to more effective healing that translates into greater strength and integrity of the anastomosis [88]. Arginine is a conditionally essential amino acid present in the diet, and supplementation promotes wound healing by increasing resistance to breakage and collagen deposition in scars [89].

### 4.2. Immune Function and Inflammation

All-trans-RA, a retinoic acid derivative, regulates transforming growth factor β (TGF-β), converting naive T cells into regulatory T cells (Tregs). This prevents autoimmunity, inhibits proinflammatory Th17 cells induced by IL-6, and promotes Treg differentiation [90]. A randomized study found vitamin A supplementation reduced serum IL-17 and TGF-β concentrations in both obese and non-obese women, along with IL-10 and IL-6, suggesting a role in immune response modulation [91].

Vitamin D is key to innate immunity because it stimulates the production of pattern recognition receptors (PRRs), antimicrobial peptides, and cytokines; it also induces the antibacterial activity of monocytes and macrophages, resulting in increased cathelicidin production [92]. In turn, this causes inhibition of the acquired immune response through inhibition of the synthesis of proinflammatory cytokines [93]. It also modulates the nuclear factor-kappaB (NF-κB) pathway by regulating the inflammatory cascade [93].

Vitamin E plays a crucial role in maintaining optimal immune function through multiple mechanisms [94]. It is an essential antioxidant that protects cell membranes from lipid peroxidation by neutralizing reactive oxygen species (ROS) and reactive nitrogen species (RNS), playing an important protective role for immune cells, which are highly vulnerable to oxidative damage due to their active metabolism and exposure to inflammatory conditions [95]. Forms of vitamin E regulate anti-inflammatory pathways through inflammatory mediators—γ- and δ-tocopherol and γ-tocotrienols—showed the most potent inhibitory activity against cyclooxygenase (COX)-2-mediated prostaglandin D_2_ and prostaglandin E_2_ formation [96]. 

A study in gilts found that dietary supplementation with 2-hydroxy-4-methylselenobutanoic acid (HMSeBA), an organic selenium source, increased serum IL-2 and immunoglobulin G while reducing IL-6 and TNF-α, thus lessening inflammation caused by ROS and RNS [97]. However, a systematic review on healthy individuals showed complex results: selenium supplementation offered small or no increases in immunoglobulins and only a slight, imprecise increase in T cells [98]. The sole positive immune effect observed was increased natural killer cell lysis (SMD = 0.48; 95% CI: 0.13–0.82) [98]. Dose–response analysis suggested immune benefits peak at ~100 µg/L plasma selenium; higher levels may be neutral or detrimental, indicating no need to exceed recommended dietary selenium intake for immune function benefits [98].

**Figure 2:**
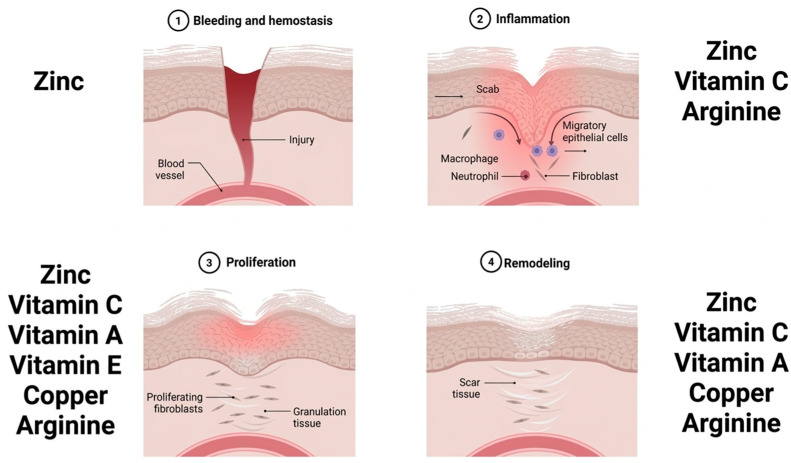
The role of nutrients in the phases of wound healing. Wound healing is a process involving consecutive and overlapping stages such as homeostasis, inflammation, proliferation or formation of new tissue, and remodeling. In each of the stages, micronutrients play an important biological role in the processes of tissue repair and immune defense. This figure examines the nutrients that act as “builders” for the wound, offering protection against infection and regulating inflammation [73,79,81,89,102].

As mentioned above, vitamin C, zinc, copper, and amino acids play a significant role in the wound-healing phases through multiple mechanisms. Other micronutrients, such as vitamins A, D, and E, as well as selenium, are involved in immune modulation (Figure 2). Vitamins C and D, as well as zinc, have the strongest evidence for immune support [99]. In surgical patients, micronutrient deficiencies compromise immune function, increase infectious complications, and negatively affect postoperative outcomes [100]. Deficiencies in nutritional status or metabolic capacity can lead to complications such as anastomotic failure, surgical site infections, delayed restoration of gastrointestinal function, and postoperative physical disability. Hospital stays may also be prolonged by these issues [101].

### 4.3. Oxidative Stress and Vascularization

ROS have a complex role in wound angiogenesis, acting as crucial intracellular signaling molecules that regulate physiological processes like vascular reactivity and angiogenesis. Their effects can be either beneficial or harmful, depending on the level and duration of oxidative stress [103]. The wound site’s germs are killed by the modest rise in ROS levels, which creates a sterile environment that promotes revascularization [104]. Only physiological levels of ROS are beneficial; excessive concentrations can be harmful [105]. Excessive ROS production leads to oxidative stress that significantly impairs wound healing [103]. High levels of ROS in endothelial cells, generated by various pathological factors or released by neutrophils, can interrupt normal endothelial function [103]. This disruption manifests as impaired endothelium-dependent vasodilation, primarily due to decreased NO bioavailability [106]. This highlights the importance of a precise redox balance for healing [105]. Intracellular ROS generation, including hydrogen peroxide (H_2_O_2_), is crucial in the regulation of angiogenesis [105]. ROS mediate angiogenic effects, such as migration, proliferation, and capillary formation, through the regulation between H_2_O_2_ and VEGF [105].

The antioxidant role of micronutrients such as vitamin C is particularly relevant, as ROS have been shown to be elevated in wounds, and animal models have demonstrated that free radical damage is associated with delayed wound healing [107]. Vitamin C is a powerful antioxidant that is effective in reducing oxidative stress damage to the skin, and when used in conjunction with vitamin E, due to its role in regenerating oxidized vitamin E, it promotes recycling and thus limits oxidative damage to cell membranes [63]. This is particularly important in the epidermis [107]. It also acts as an antioxidant, protecting immune cells against ROS produced during the phagocytosis process of the immune system in the degradation of pathogens and fungi [107].

Zinc exerts its antioxidant action by competing with ions that catalyze the production of highly damaging ROS, such as iron and copper, by binding to cell membranes and proteins, reducing the formation of ROS [108]. It also binds to the thiol (-SH) groups of biomolecules, protecting them from oxidation, and increases the activity of catalase, glutathione (GSH), and superoxide dismutase (SOD) while simultaneously reducing the activity of pro-oxidant enzymes such as inducible nitric oxide synthase (iNOS) and NADPH oxidase. It also induces the expression of metallothionein, which acts as a hydroxyl radical (HO•) scavenger [108].

Selenium’s well-known antioxidant activity, protecting cells from oxidative damage, is crucial for maintaining immune function, helping to regulate ROS and RNS, and preventing excessive production that can lead to lipid peroxidation, DNA damage, and protein degradation, all of which profoundly affect immune function [109]. A defining characteristic of innate immunity is its rapid response to infection [109]. Leukocytes kill pathogens by generating ROS during a biochemical reaction called “oxidative burst”, a process regulated in part by selenoproteins. In turn, selenium’s antioxidant mechanisms control the excessive production of ROS, which can damage host tissues [109].

## 5. Link Between Micronutrient Deficiencies and Fistula Risk

### 5.1. Evidence from Gastrointestinal and Colorectal Surgery

In laboratory studies with rats, the effect of micronutrients on anastomotic leakage was studied. In a study with rats with food restriction, supplementation with 100 mcg/kg/day before colon resection decreased the intra-abdominal adhesion index and raised collagen density and hydroxyproline concentration [110]. In another laboratory study, supplementation with 100 mcg/kg/day or 200 mcg/kg/day in rats with colonic transection and anastomoses enhanced anastomotic strength, inflammatory reaction, and collagen buildup [111]. Regarding zinc supplementation, laboratory studies with rats with colonic anastomosis did not show clear results [112,113].

Several observational studies in colorectal surgery have shown that preoperative malnutrition is associated with an increased risk of anastomotic leak, while there was no difference in BMI (with leak 23:39 ± 3:14 kg/m^2^ vs. no leak 23:03 ± 3:56 kg/m^2^; P 0.44) [114]. A recent prospective study has provided direct evidence linking a specific folic acid deficiency with the risk of anastomotic leak [115]. This study showed that low preoperative folic acid levels are significantly associated with the presence of sarcopenia in patients undergoing surgery for colorectal cancer; only 2.4% (*n* = 5) of the non-sarcopenic individuals showed low FA levels in their preoperative blood tests, compared to 60% (*n* = 24) of the 40 patients with radiological sarcopenia [115]. The mean BMI in both groups was 27.25 kg/m^2^ (5.27) vs. 26.99 kg/m^2^ (5.11) [115]. With 40.0% (*n* = 16) of patients in the sarcopenia group and just 0.5% (*n* = 1) in the non-sarcopenia group, the rate of AL was significantly greater (*p* < 0.01) [115]. Also, low vitamin D levels were linked to increased anastomotic leakage in a prospective cohort study involving 535 patients who had right colon cancer surgery with stapled anastomosis [116]. Finally, in an observational trial with 228 oncologic patients that underwent a surgical procedure, it was observed that patients with low vitamin C levels had higher anastomotic leak (*p* = 0.049) and higher blood loss (*p* = 0.006) [117]. 

**Figure 3:**
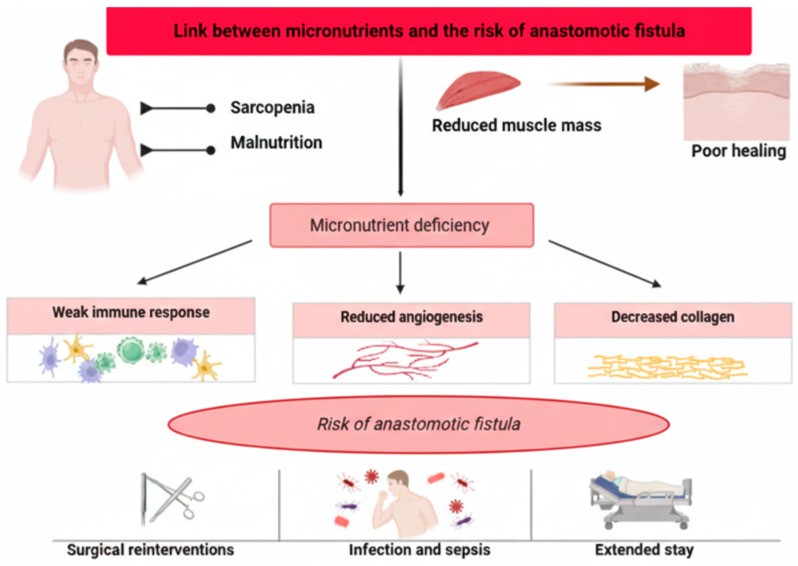
Linking micronutrient deficiencies to the risk of postoperative fistula. The figure summarizes the mechanisms by which nutritional status and specific micronutrient deficiencies influence the risk of postoperative fistula. Preoperative malnutrition and folic acid deficiency are associated with sarcopenia and reduced muscle mass, which compromises tissue healing. Micronutrient deficiencies (folic acid, vitamins D, A, and C, iron, zinc, and selenium) affect key tissue repair processes, including the immune response, angiogenesis, and collagen synthesis. These pathophysiological alterations increase the risk of anastomotic leak and are associated with higher rates of infection, sepsis, the need for reinterventions, and prolonged hospital stays [115,118,120]. Created in BioRender.com.

From a pathophysiological perspective, multiple micronutrients and macronutrients play an essential role in tissue healing [118]. Deficiencies in macronutrients, proteins, carbohydrates, and fats compromise key processes such as angiogenesis and collagen production [118]. Likewise, certain amino acids, such as glutamine and arginine, vitamins A, C, and D, and minerals, such as zinc, selenium, and iron, act as essential cofactors in the different phases of wound healing [118]. These processes are critical for proper wound healing and postoperative recovery, preventing potential complications [119]. Figure 3 summarizes the pathophysiological processes.

Postoperative fistula, including anastomotic leak, is one of the most serious complications in gastrointestinal surgery and is associated with a significant increase in morbidity and mortality, reoperations, healthcare costs, and prolonged hospital stay [120]. In colorectal surgery, anastomotic leak has been extensively studied and is recognized as a multifactorial event influenced by various local and systemic factors [121]. Its incidence varies between 1% and 19%, being more frequent in the rectal area [120]. Among the systemic factors, the patient’s nutritional status has become an important determinant. Nutritional assessment and conditioning of patients during the perioperative period are of particular importance [114]. Furthermore, several studies have confirmed that providing nutritional support to those suffering from preoperative malnutrition reduces the occurrence of anastomotic leak and other complications [114]. However, the specific role of micronutrient deficiencies as independent determinants of fistula risk has been underestimated, especially in bariatric and metabolic surgery [24].

### 5.2. Observational Studies and Case Reports in Bariatric Surgery

Direct clinical evidence associating micronutrient deficiencies with the incidence of postoperative fistulas in bariatric surgery is limited [24]. Most observational studies in bariatric surgery focus on long-term metabolic and nutritional outcomes, without analyzing early surgical complications such as fistulas [122]. In large surgical series, nutritional variables are usually limited to body mass index, nutritional screening, or serum albumin [114]. The limited evidence of detailed micronutrient data prevents establishing clear associations with fistula risk [24]. Case reports occasionally describe persistent fistulas or healing complications in patients with severe malnutrition [123]. However, in most of these reports, the nutritional assessment is retrospective and not standardized [123]. This methodological limitation reduces the ability to establish causal relationships between micronutritional deficiencies and postoperative fistulas [123].

It has been observed that patients with a high body mass index present with micronutrient deficiencies before bariatric surgery [24]. A cross-sectional study with obese patients with a mean BMI of 42.8 kg/m^2^ who underwent bariatric surgery showed that 85.5% of patients had at least one micronutrient deficit, and the most common condition was vitamin D insufficiency (74.5%) [24]. This inadequate nutritional profile may predispose patients to a poor response to surgical stress [24]. Following bariatric surgery, especially in procedures with a malabsorptive component such as Roux-en-Y gastric bypass, micronutrient deficiencies can persist or worsen [122]. Another observational study with patients undergoing bariatric surgery with a BMI of 44 ± 9 kg/m^2^ has documented sustained deficiencies in iron, ferritin, folate, vitamin B12, vitamins D and A, as well as minerals, such as zinc, copper, and selenium, during postoperative follow-up [122]. These deficiencies are observed even in patients receiving standard supplementation [123]. Patients with higher degrees of obesity (mean BMI 42.4 ± 4.7 kg/m^2^) and women should be given greater consideration [124]. 

In case series with small sample sizes and expert opinions, it is suggested that micronutrient deficiencies may contribute to the development of fistulas in bariatric surgery [122]. It has been proposed that deficiencies in specific nutrients such as zinc, iron, folate, and vitamins A and C could interfere with collagen synthesis and angiogenesis [115,118]. 

### 5.3. Limitations and Gaps in Current Research

While fistula formation is often attributed to technical failures in suturing or stapling, from a biological perspective, the persistence of an abnormal communication reflects a defect in the healing cascade. The mechanical closure provided by the surgeon must be followed by proliferation and extracellular matrix remodeling phases, processes that are strictly dependent on the availability of micronutrients such as zinc, copper, and vitamins A and C. A key limitation of the current literature is the lack of systematic reporting of micronutritional status in bariatric surgical outcome studies [122]. As previously described, micronutrient deficiencies are rarely included as predictive variables in fistula risk models [120]. This omission contributes to micronutrient deficiencies remaining an underestimated risk factor [24].

Currently, there are no standardized protocols for micronutrient assessment in patients who develop postoperative fistulas [123]. Another aspect to highlight as a gap is that the studies are not designed to show differences in micronutrient deficiency and the different degrees of obesity. Furthermore, there is no consensus on what should be measured or at what point in the perioperative period [123]. So far, the publications detailing risk factors include extensive measurement of variables and different selections of micronutrients, which prevents a direct association between the micronutrients and the development of fistulas [120,123,125].

## 6. Prevention and Management Strategies

### 6.1. Preoperative Screening and Correction

According to the 1991 National Institutes of Health (NIH) consensus statement on Gastrointestinal Surgery for Severe Obesity, all patients potentially undergoing metabolic surgery should be evaluated by a multidisciplinary team including medical, surgical, psychiatric, and nutritional experts [126]. Currently, there is sufficient evidence to affirm that perioperative nutritional management plays a very important role [127,128,129]. Therefore, the recommendations of the various guidelines consulted are unanimous: they recommend a complete nutritional assessment and perioperative dietary recommendations, as these are fundamental for controlling metabolic syndrome, reducing preoperative malnutrition, and correcting micronutrient deficiencies, thereby decreasing the incidence of postoperative complications and promoting a rapid recovery [127,128,129].

Both the American Society for Metabolic and Bariatric Surgery (ASMBS) and the International Federation for the Surgery of Obesity and Metabolic Disorders (IFSO), in their 2022 update of indications, and the 2022 Clinical Practice Statement (CPS) of the Obesity Medicine Association (OMA) on bariatric surgery, gastrointestinal hormones, and the microbiome, propose a preoperative nutritional assessment that should include: medical history (comorbidities, surgeries, allergies, exploration of legal and illegal substance use, sleep quality, and mental health), review of medication use that could potentially increase weight, history of current weight and body composition (via bioimpedance or DEXA), eating behaviors, food insecurity, physical activity, and a complete laboratory test with assessment of specific micronutrients according to the type of surgery to be performed [127,130]. The Enhanced Recovery After Surgery (ERAS) guide on this topic, published in 2021, reinforces the importance of nutritional assessment in the preoperative period and the indication for weight loss through very low-calorie and low-fat diets because it would reduce liver size, although the most current guidelines no longer place so much emphasis on this point [128].

The main point to consider when evaluating and supplementing micronutrients is knowing the absorption site of each one in the gastrointestinal tract in order to more accurately assess the risk of post-surgical deficiency [130]. Micronutrient deficiency in patients with morbid obesity has a high incidence and develops as a consequence of multiple factors related to the pathophysiology of obesity [26]. 

Preoperative measurement of iron, vitamin B12, folate, vitamin D, calcium, and thiamine is recommended for all patients, along with their respective supplements, to assess metabolism and reserves. Copper, zinc, and vitamin A should be added if malabsorptive procedures are performed [60,131,132]. Intervention thresholds in each case will depend on the severity of the depletion, and the corresponding loading protocol for each micronutrient should be implemented immediately to correct the deficiency by the time of surgery [131].

### 6.2. Tailored Supplementation Protocols

Micronutrient supplementation will be recommended for all patients from the immediate postoperative period, including multivitamins containing thiamine, zinc, copper, folic acid, and selenium, specifically formulated for this type of surgery. These supplements should provide more than 200% of the recommended daily intake. Furthermore, the medical protocol should be followed when prescribing vitamin D (3000 IU), calcium citrate (1200–1500 mg), iron (45–60 mg), and sublingual or injectable vitamin B12 [59,133]. Supplementation with fat-soluble vitamins is not indicated on a regular basis; the need should be evaluated in each case according to the serum levels found, which are recommended to be monitored [58]. Table 1 summarizes the supplementation recommendations for these patients and compares them with the recommendations for other digestive tract surgeries.

Currently, personalized supplementation protocols are recommended, initially based on the risk of deficiency due to the type of surgery and subsequently based on each individual’s laboratory values during follow-up appointments at one month, and then every three months until one year, every six months for the second year, and then annually [22,134]. The dosage protocol for each surgical technique takes into account the increased risk of malabsorption associated with the technique [58,59,133]. 

**Table 1:** Micronutrient requirements in bariatric surgery vs. other gastrointestinal surgeries.

Nutrient	Recommended Dosage		Quality of Evidence	
	Bariatric Surgery	Others GI Surgery’s	Bariatric Surgery	Others GI Surgery’s
Vitamin D	2000–4000 UI/day	Cover RDA or correct deficiency	Moderate [128]	Low [128]
Folate	400–800 ug/day *	Cover RDA	Moderate [128]	Low [128]
Vitamin C	100–200 mg/day *	Cover with daily food, except in cases of deficiency or complex healing	Moderate [128]	Low [128]
Zinc	8–15 mg/day *	No routine supplementation, except in cases of chronic diarrhea or fistulas	Low-Moderate [128]	Low [128]
Selenium	30–50 ug/day *	Not specific evidence	Low [128]	Low [128]
Iron	45–60 mg/day	Cover RDA and supplementation in elective surgery with preoperative anemia	High [128]	Low-Moderate [128]
Protein	1–1.5 g/kg **/day	Not routinely; assess according to surgery	Moderate [128]	Low [128]

* cover from the multivitamin supply. ** kilogram of adjusted body weight. The table summarizes the recommendations for micronutrient supplementation according to the type of surgical technique used, thus considering the impossibility for the new anatomy to optimally absorb these nutrients. mg: milligram; mcg: microgram; UI: international unit. GI: gastrointestinal RDA: recommended dietary allowance [56,57,133,135,136,137,138].

### 6.3. Timing: Preoperative Correction vs. Postoperative Maintenance

Level-adjustment protocol, where the dose is personalized according to the blood level measured at that moment, and action is taken accordingly [59]. When prescribing supplementation, the current post-surgical phase should be considered when selecting the supplement consistency to promote tolerance and adherence, especially in patients who experience nausea. It is suggested that for the first 15 days, it be administered in liquid form and then as chewable tablets [59].

### 6.4. Multidisciplinary Monitoring and Follow-Up

Primary care, along with nutritional monitoring, will form the backbone of long-term follow-up [139]. Their care will focus on monitoring the patient’s overall health status, from nutritional to psychosocial aspects [139], and it is responsible for guiding the patient through the various follow-up appointments and check-ups [139]. The primary care physician is important in helping patients develop a realistic awareness of weight gain and plateaus; also, emphasizing improvements in overall health rather than changes in BMI can contribute to broader health outcomes and quality of life [139]. Their efforts are crucial in lowering irrational expectations, lowering feelings of guilt or shame, and avoiding compensatory behaviors that may result in eating disorders [139]. 

It is essential that bariatric patients undergo rigorous postoperative monitoring of their vitamin and micronutrient levels to identify early deficiencies that could compromise recovery, even every three months during the first year of surgery [59]. In this clinical context, it is crucial to note that serum vitamin B12 levels may appear within the normal range (200–400 pg/mL) despite a real cellular deficiency [59,140]. Therefore, in bariatric patients at risk of healing complications, measuring methylmalonic acid (MMA) and homocysteine is preferable to confirm metabolic status [59,140]. Similarly, vitamin D assessment should consider that serum 25(OH)D can be influenced by hemodilution and postoperative inflammation; thus, more stringent thresholds should be applied to ensure adequate bone and tissue health [59]. The nutritionist’s role in follow-up is crucial [141]. They must collaborate with the patient to ensure they meet their macronutrient and micronutrient requirements while simultaneously providing education that encourages a change in diet quality and the adoption of healthy habits, thus preventing excessive muscle loss and weight regain [141,142]. Long-term nutritional follow-up has been shown to contribute to successful weight loss regardless of age, sex, and BMI and to improve patient adherence [143]. In patients experiencing complications, such as nausea, dumping syndrome, and digestive symptoms, and signs related to malabsorption from the surgical technique, among others, special attention should be paid to dietary modifications, and medical management will be necessary. Therefore, a multidisciplinary approach is once again essential at this stage [143,144,145]. Finally, in the case of surgeons, they will lead the team, reinforcing with the patient the importance of and commitment to long-term follow-up [143]. They will address medium- and long-term complications that require their intervention, including those requiring revision surgery to ensure the success of the treatment, and will supervise the actions of the rest of the team [143].

In addition to the follow-up group, there need to be objective tools that allow us to have a correct evaluation of the patient. Monitoring and assessing adherence require a multidisciplinary approach [146]. It should be holistic, combining structured follow-up by the treating healthcare team, validated questionnaires, and currently available technological alternatives [146]. The healthcare team’s follow-up will be guided by current recommendations and the adaptation of the medical protocol adopted in each case [146]. 

Many studies using self-administered questionnaires show inconsistencies in methodology or in the definitions of adherence, and are therefore unreliable for making generalized recommendations [146]. The Eating Behavior Questionnaire after Bariatric Surgery (EBBS) addresses adherence to dietary and lifestyle changes [146,147]. It contains 11 scored items, and higher scores correlate with better long-term weight loss outcomes [146,147]. To assess adherence to vitamin and mineral supplementation, the Medication Adherence Report Scale (MARS-5) is used [146,148]. This self-administered questionnaire consists of five questions about missed doses, dose changes, discontinuation, omission, and reduction in medication [146,148]. It has been used in studies to evaluate the intake of oral supplements [146,148]. Currently, the advancement of digital tools necessitates their inclusion among the available resources [149]. A systematic review and meta-analysis published in 2024, which evaluated five studies with a total of 2210 patients, found that the intervention group (receiving telemedicine assistance) showed a 10% increase in total weight loss and a 22% reduction in excess weight loss [149]. Also, emergency room visit rates showed a non-statistically significant decrease; therefore, while statistical superiority is not demonstrated, we should consider conducting further studies as it shows promise [149]. It is necessary to generate scientific evidence to standardize recommendations for assessing adherence [146].

### 6.5. Summary of Recommendations

- Preoperative Screening: All candidates for metabolic surgery must be evaluated by a multidisciplinary team. It is recommended to measure baseline levels of iron, vitamin B12, folate, vitamin D, calcium, and thiamine for all patients.
- Targeted Assessment: For patients undergoing malabsorptive procedures, screening for copper, zinc, and vitamin A should be added.
- Correction of Deficiencies: Identified deficiencies must be addressed with specific loading protocols and supplements before surgery to assess metabolism and reserves.
- Absorption Awareness: Clinicians must consider the specific absorption site of each micronutrient in the gastrointestinal tract to accurately assess the risk of postoperative deficiency.
- Personalized Supplementation: Post-surgical supplementation should follow a level-adjustment protocol, where doses are personalized based on blood levels measured at that specific moment.
- Tolerance and Adherence: During the first 15 days post-surgery, supplements should be administered in liquid form, transitioning later to chewable tablets to promote tolerance and long-term adherence.
- Multidisciplinary Long-term Follow-up: Primary care and nutritional monitoring are the backbone of recovery. The team (surgeons, primary care physicians, and nutritionists) must work together to monitor nutritional status, weight gain, and psychosocial aspects.
- Standardized Requirements: According to the summarized data (Table 1), key daily requirements include:
  ○ Vitamin D: 2000–4000 IU.
  ○ Folate: 400–800 µg.
  ○ Iron: 45–60 mg.
  ○ Protein: 1–1.5 g/kg (adjusted weight).

### 6.6. Clinical Perspectives

Despite the clinical relevance of the mechanisms described, this review has limitations. Most available evidence stems from observational studies and animal models, with a lack of large-scale randomized controlled trials (RCTs) specifically linking preoperative micronutrient levels to fistula rates. Furthermore, the heterogeneity in surgical techniques and supplementation protocols across studies makes it difficult to establish universal threshold levels for every micronutrient.

From a clinical standpoint, we suggest that bariatric programs implement mandatory comprehensive nutritional screenings at least 3 months prior to surgery. Future research should focus on “pre-habilitation” protocols, investigating whether aggressive intravenous correction of specific deficits (such as zinc and vitamin C) in the immediate preoperative period can significantly reduce the incidence of leaks in high-risk patients. This shift toward a multidisciplinary metabolic focus is essential for improving the safety profile of bariatric and metabolic procedures.

## 7. Future Directions

Recent evidence suggests that micronutrient deficiencies are present in obese patients both before and after bariatric surgery and that these imbalances can persist or worsen following the surgical intervention [22]. Daniela L. González-Sánchez et al., in their systematic review of 27 studies with participants from Latin America, found a high prevalence of micronutrient deficiencies both before and after bariatric surgery, with vitamins D and A and zinc being the most prevalent [150]. As mentioned in former sections, such deficiencies could contribute to a higher risk of postoperative complications, including fistulas, due to their fundamental role in tissue healing, immune response, and intestinal barrier integrity [119]. Although current guidelines provide useful tools, most are based on low-level evidence, highlighting the need for more robust research [22]. While there are associations between micronutrient deficiencies and adverse clinical outcomes in gastrointestinal surgery, specifically bariatric surgery, there is no conclusive evidence on how to prevent and treat micronutrient deficiencies before surgery to optimize patient outcomes [151]. Therefore, controlled clinical trials with specific perioperative nutritional interventions are needed to assess whether correcting nutritional status would reduce the incidence of postoperative complications [151]. In parallel, the development and validation of reliable biomarkers for subclinical deficiency and risk of complications represents a relevant methodological frontier [151].

Furthermore, it has been suggested that bariatric surgery may induce changes in the composition and function of the gut microbiome, which have been linked to both micronutrient absorption and postoperative hormonal and metabolic regulation [152,153,154]. Microbiome alterations could, in themselves, influence healing processes, intestinal permeability, and systemic inflammation—mechanisms related to postoperative complications [153]. In the study by Suárez-Sánchez et al. on severely obese patients undergoing malabsorptive bariatric surgery, microbial abundance patterns changed after surgery and were linked to metabolic pathways related to micronutrient metabolism [155]. Additionally, it describes a possible correlation between the intake of micronutrients, such as magnesium and thiamine, and changes in bacterial species post-surgery, specifically *Coprococcus* sp., an SCFA-producing bacterium, which reinforces the idea of a complex interaction between bariatric surgery, nutrients, and microbiota [155]. These results suggest that bariatric surgery and nutrients can influence microbiome composition, opening a potential avenue for optimizing recovery through dietary strategies and thus minimizing risks such as micronutrient deficiencies and postoperative complications [155]. In this way, modulating the gut microbiota, even with the use of probiotics, could reduce bacterial overgrowth, optimize the synthesis and availability of micronutrients, and potentially improve the overall nutritional status of bariatric patients [152]. Evidence linking gut microbiota to bariatric surgery has grown exponentially in the last decade, with an increasing number of studies using more integrative approaches that explore metabolic, inflammatory, and nutritional mechanisms; however, short-term observational studies predominate, with methodological heterogeneity, making a greater number of longitudinal trials integrating microbiota, micronutrients, and relevant clinical outcomes necessary [154,156,157]. This suggests that, while the microbiota is recognized as a key factor in the effects of bariatric surgery, its role in the development of nutritional deficiencies and postoperative complications remains insufficiently characterized, reinforcing the need for longitudinal studies that integrate microbiological, nutritional, and clinical variables [154,156,157]. Integrating microbiological data with nutritional and surgical profiles in advanced analytical models could identify at-risk patients, enabling personalized interventions [158].

**Figure 4:**
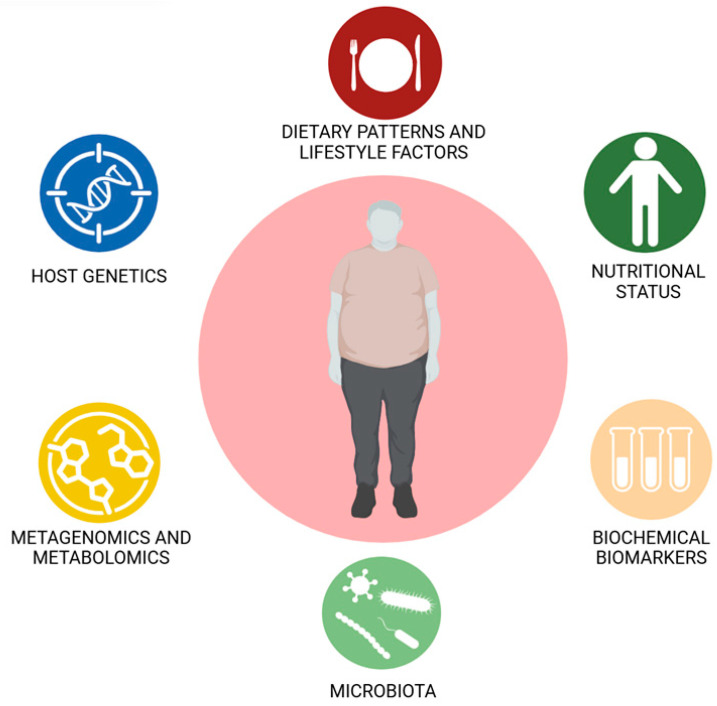
Personalized nutrition: a multidimensional concept that seeks to integrate and interpret the complex interactions between dietary patterns, lifestyle factors, nutritional status, host genetics, biochemical biomarkers, metabolic characteristics, and omic profiles, including metagenomics and metabolomics [102,159]. This information enables a more precise characterization of individual variability in nutrient requirements, metabolic responses, and susceptibility to nutritional deficiencies and postoperative complications [102,159]. This approach facilitates the personalization of nutritional interventions aimed at optimizing micronutrient adequacy, metabolic regulation, and gut microbiota balance, thereby contributing to improved prevention and management of chronic diseases and surgery-related complications, including those affecting postoperative recovery and long-term outcomes [102,159].

Precision nutrition, or personalized nutrition, takes into account genetic, epigenetic, microbiota, nutritional status, and individual clinical characteristics [102,159]. This approach integrates individual information on nutritional status, lifestyle factors, dietary patterns, host genetics, biochemical biomarkers, metabolic characteristics, omic profiles, metagenomics, and metabolomics, among others, with the aim of stratifying patients according to their risk of complications and personalizing perioperative intervention strategies [102,158,159] (Figure 4). Personalized nutrition has the potential to prevent deficiencies that could compromise the physiological response to surgical trauma, tissue healing, and anastomotic integrity, reducing the risk of fistulas and other severe complications [102,159]. Future studies validating algorithms and demonstrating clinical benefits will be crucial to justifying the implementation of personalized nutrition in bariatric surgery [102,159].

Overall, evidence causally linking micronutrient deficiencies to complications such as fistulas and anastomotic leaks in bariatric surgery patients is still limited. Future research should prioritize interventional clinical trials, robust prospective cohorts, and the development of integrative predictive models that allow not only for understanding the underlying mechanisms but also for implementing personalized prevention and management strategies for these patients.

## 8. Conclusions

The evidence presented in this review highlights a critical yet frequently underestimated aspect of bariatric and metabolic surgery: the determining role of micronutrients in preventing serious complications such as postoperative fistulas. Although obesity is commonly associated with caloric excess, the nutritional status of these patients is often marked by significant deficiencies in essential vitamins and minerals. These conditions can be drastically exacerbated following surgical intervention due to malabsorption, restricted intake, and anatomical and physiological alterations of the gastrointestinal tract.

Vitamins and trace elements act as indispensable cofactors in collagen synthesis, fibroblast proliferation, and angiogenesis, all of which are vital processes for ensuring the structural integrity of gastrointestinal anastomoses. A preexisting or uncorrected deficiency in these micronutrients can impair tissue repair and healing capacity, thereby increasing patient vulnerability to anastomotic leaks and fistula formation. Such complications carry high morbidity and mortality rates, leading to increased reinterventions and a substantial contribution to increased healthcare costs. Furthermore, the impact of micronutrients extends to immune modulation and oxidative stress regulation. Consequently, the perioperative management of patients with obesity is essential to prevent these kinds of complications. 

Despite strong biological plausibility, direct clinical evidence linking micronutrient deficiencies to postoperative fistulas in bariatric surgery remains limited. Micronutrient status is still infrequently incorporated into surgical risk stratification models, representing a notable gap in current clinical practice and research. Although surgical technique remains the most immediate determinant of success, pre- and postoperative micronutrient status represents a critical modifiable factor. We propose that nutritional optimization is not merely supportive but a fundamental biological component required to minimize fistula risk in patients with severe obesity. Future studies should prioritize well-designed prospective and interventional trials to clarify these associations and to determine whether targeted micronutrient supplementation can effectively reduce the incidence of anastomotic complications. Improving surgical outcomes in this high-risk group may need an integrated and customized strategy for perioperative nutritional assessment and management.

## Data Availability

No new data were created or analyzed in this study.

## Abbreviations

The following abbreviations are used in this manuscript:

25(OH)D	25-hydroxyvitamin D
ASMBS	American Society for Metabolic and Bariatric Surgery
BMI	Body Mass Index
COX	Cyclooxygenase
CPS	Clinical Practice Statement
DEXA	Dual-Energy X-Ray Absorptiometry
EBBS	Eating Behavior after Bariatric Surgery Questionnaire
ECM	Extracellular Matrix
ERAS	Enhanced Recovery After Surgery
GB	Gastric Bypass
GLP-1	Glucagon-Like Peptide-1
GIP	Gastric Inhibitory Polypeptide
GSH	Glutathione
H_2_O_2_	Hydrogen Peroxide
HMSeBA	2-hydroxy-4-methylselenobutanoic acid
HO•	Hydroxyl Radical
IFSO	International Federation for the Surgery of Obesity and Metabolic Disorders
IL	Interleukin (e.g., IL-6)
iNOS	Inducible Nitric Oxide Synthase
MMPs	Matrix Metalloproteinases
mVAD	Moderate Vitamin A Deficiency
NF-κB	Nuclear Factor-KappaB
NO	Nitric Oxide
NOS	Nitric Oxide Synthase
OMA	Obesity Medicine Association
oVAD	Overall Vitamin A Deficiency
PD	Pancreaticoduodenectomy
PDGF	Platelet-Derived Growth Factor
PRRs	Pattern Recognition Receptors
RDA	Recommended Dietary Allowance
RNS	Reactive Nitrogen Species
ROS	Reactive Oxygen Species
RYGB	Roux-en-Y Gastric Bypass
SCFA	Short-Chain Fatty Acids
SG	Sleeve Gastrectomy
SIBO	Small Intestinal Bacterial Overgrowth
SOD	Superoxide Dismutase
TGF-β	Transforming Growth Factor β
Th	T-Helper (e.g., Th2, Th9, and Th22)
TNFα	Tumor Necrosis Factor Alpha
Tregs	Regulatory T Cells
VEGF	Vascular Endothelial Growth Factor
ZFX	Zinc-Finger X-Linked Protein
